# The twin-arginine translocation system is vital for cell adhesion and uptake of iron in the cystic fibrosis pathogen *Achromobacter xylosoxidans*

**DOI:** 10.1080/21505594.2023.2284513

**Published:** 2023-11-16

**Authors:** S. M. Hossein Khademi, Cecilia Sahl, Lotta Happonen, Åke Forsberg, Lisa I. Påhlman

**Affiliations:** a Department of Clinical Sciences Lund, Division of Infection Medicine, Lund University, Lund, Sweden; b Department of Molecular Biology, Umeå University, Umeå; c Division of Infectious Diseases, Skåne University Hospital Lund, Lund, Sweden; d Wallenberg Centre for Molecular Medicine, Lund University, Sweden, Sweden

**Keywords:** Twin-arginine translocation, bacterial protein secretion, iron metabolism, *achromobacter xylosoxidans*, infection, cystic fibrosis

## Abstract

*Achromobacter xylosoxidans* is an emerging pathogen that causes airway infections in patients with cystic fibrosis. Knowledge of virulence factors and protein secretion systems in this bacterium is limited. Twin arginine translocation (Tat) is a protein secretion system that transports folded proteins across the inner cell membranes of gram-negative bacteria. Tat has been shown to be important for virulence and cellular processes in many different bacterial species. This study aimed to investigate the role of Tat in iron metabolism and host cell adhesion in *A. xylosoxidans*. Putative Tat substrates in *A.* *xylosoxidans* were identified using the TatFind, TatP, and PRED-Tat prediction tools. An isogenic *tatC* deletion mutant (ΔtatC) was generated and phenotypically characterized. The wild-type and ΔtatC *A.* *xylosoxidans* were fractionated into cytosolic, membrane, and periplasmic fractions, and the expressed proteome of the different fractions was analysed using liquid chromatography-mass spectrometry (LC-MS/MS). A total of 128 putative Tat substrates were identified in the *A.* *xylosoxidans* proteome. The ΔtatC mutant showed attenuated host cell adhesion, growth rate, and iron acquisition. Twenty predicted Tat substrates were identified as expressed proteins in the periplasmic compartment, nine of which were associated with the wild type. The data indicate that Tat secretion is important for iron acquisition and host cell adhesion in *A.* *xylosoxidans*.

## Introduction

Cystic fibrosis (CF) is a monogenetic disease characterized by persistent microbial infection of the airways, leading to a progressive decline in lung function [1]. The gram-negative rod *Achromobacter xylosoxidans* is an emerging CF pathogen that infects approximately 5% of individuals with CF [2–4]. Persistent *Achromobacter* infections are associated with poor lung function [2,5], but very little is known about the virulence factors of this opportunistic pathogen. During persistent infection, CF pathogens, such as *Pseudomonas aeruginosa* and *A. xylosoxidans* adapt to the host environment via genetic evolution to promote chronic colonization [6,7].

Bacteria utilize various protein secretion systems that are important for bacterial virulence [8], and different secretion systems are likely to be involved at different stages of infection. While Type 3 secretion is believed to be important for the early phase of *P. aeruginosa* infection in CF lungs [9,10], the twin-arginine translocation system (Tat) has been implicated in cellular processes relevant to persistent infection, such as iron acquisition [11]. In gram-negative bacteria, the Tat system transports proteins across the inner membrane to the periplasmic space. Proteins are translocated in a fully folded state, which allows them to be transported together with co-factors or as hetero-oligomeric complexes [12]. The Tat complex consists of three membrane-bound subunits: TatA, B, and C. Tat substrates are targeted to the Tat complex by an N-terminal signal peptide containing a conserved twin-arginine motif [13]. Tat secretion is vital for bacterial virulence in many different human pathogens [14–18], including the CF pathogen *P. aeruginosa* [19]. As the Tat system is involved in the secretion of proteins with diverse functions, the role of Tat depends on the nature of the transported Tat substrates. Consequently, Tat secretion has been implicated in various cellular processes such as cell growth, motility, and iron acquisition [11].

Both Type 3 secretion and Tat are present in *A. xylosoxidans* based on data from published genomes [20,21]. The aim of this study was to characterize the role of Tat secretion in *A. xylosoxidans* and to establish whether the system is important for the establishment and persistence of infection. This was done by generating a mutant deficient in Tat secretion and characterizing the proteomes of the wild type and mutant to elucidate the mechanistic backbone of important phenotypes related to Tat secretion.

## Materials and methods

### Bacterial strains, plasmids and growth conditions

The bacterial strains, plasmids, and primers used in the present study are listed in Table 1. Luria-Bertani (LB) broth was used for routine preparation of bacterial cultures at 37 °C. For maintenance of plasmids in *Escherichia coli*, 25 μg ml^−1^ of chloramphenicol was used. To select the deletion strain of *A. xylosoxidans*, 150 μg ml^−1^ of chloramphenicol was used.

**Table 1.** Bacterial strains, plasmids and primers used in this study.

Strains, plasmids or Primers			Relevant genotype, phenotype or description
**Strains**			
	***E. coli***		
	DH5alpha λpir		DH5alpha lysogenized with λ-pir phage, used for maintenance of recombinant plasmids
	***A. xylosoxidans***		
	LC1373		Wild-type *A. xylosoxidans* strain isolated from the sputum of a person with cystic fibrosis in Sweden
	LC1373-ΔtatC		LC1373 with a 744 bp deletion in the *tatC* gene
**Plasmids**			
	pDM4		Cm^R^ *sacB*^+^ R6K *ori*, mobRP4, allelic replacement vector
	pDM4-ΔtatC		pDM4 with a 609-bp BgIII-XbaI fragment upstream and a 691-bp XbaI-XhoI fragment downstream tatC
**Primers**			
	tatC1-F-*BgI*II		5’-ATATAGATCTCAGCCTCACTGAACTGATGGT-3’
	tatC1-R-*Xba*I		5’-ATATTCTAGATTCTTGGGAGGCGTCCTG-3’
	tatC2-F-*Xba*I		5’-ATATTCTAGACTGACCGAACGCCACTGA-3’
	tatC2-R-*Xho*I		5’-ATATCTCGAGAAGCCGATGATGGATACGTCG −3’

### Identification of putative tat substrates

To identify putative Tat substrates, three available *in silico* prediction tools, TatFind, TatP, and PRED-Tat, were used as previously described [22] for *A. xylosoxidans* SOLR10 proteome (NCBI accession no. CP025774). When Tat substrates were identified using TatP [23] (https://services.healthtech.dtu.dk/service.php?TatP-1.0), the regular expression parameter was changed to [RK][KR][FGAVML][LITMVF] to include possible alternative conformations. The minimum positive (Yes) score for the selection of the Tat substrate was set to 1. The “Original model” was used for identification using PRED-Tat [24]. TatFind [25] v1.4 (http://signalfind.org/tatfind.html) was used with default parameters. Tat substrates identified by at least two of the three tools were considered putative substrates for further analysis.

### In-frame deletion of *tatC* gene

A 609-bp fragment upstream and inclusive of 24 bp within *tatC* coding region was amplified from *A. xylosoxidans* LC1373 genomic DNA by PCR using tatC1-F-*BgI*II and tatC2-R-*Xba*I primers (Table 1) and digested with *BgI*II and *Xba*I restriction enzymes. A 691-bp fragment downstream, inclusive of 18 bp within *tatC* coding region, was amplified by PCR using tatC2-F-*Xba*I and tatC2-R-*Xho*I primers and digested with *Xba*I and *Xho*I restriction enzymes. These fragments were ligated into *BgI*II- and *Xho*I digested pDM4 plasmids and electroporated into *E. coli* DH5alpha λpir. The plasmid sequence was verified by Sanger sequencing (Eurofins, Germany). The resulting plasmid, pDM4-ΔtatC, was selected on LB agar plates containing 25 μg ml^−1^ chloramphenicol. The plasmid was transformed into *A. xylosoxidans* LC1373 by electroporation as previously described [26]. Merodiploid mutants were selected by plating on LB agar plates containing 150 μg ml^−1^ chloramphenicol. Colonies were streaked on 10% (w/v) sucrose-LB plates without NaCl several times until they became susceptible to chloramphenicol. Sucrose-resistant/chloramphenicol-susceptible colonies were streaked on sucrose-LB plates and deletion mutants were verified by PCR and Sanger sequencing at Eurofins.

### Fractionation of the *A.* *xylosoxidans* proteome

The secreted, periplasmic, cytosolic, and membrane fractions of *Achromobacter* were separated using osmotic shock fractionation as previously described [27]. *A. xylosoxidans* wild-type and ΔtatC mutants were grown to mid-exponential phase (OD_600_ approx. 0.3–0.4) in 50 ml of ABT minimal medium [28] supplemented with 0.5% (w/v) casamino acids and 0.5% (w/v) glucose (Merck) (ABTGC) with or without 10 µM Fe^3+^. The two cultures were normalized to each other by transferring a volume of $\frac{8\mathrm{ml}}{\mathrm{OD}\left[\mathrm{opticaldensity}\right]\mathrm{at}600\mathrm{nm}}$ to a new tube. The cells were pelleted by centrifugation at 4800 g for 15 min at 4 °C, resuspended in 850 µL phosphate buffered saline (PBS), transferred to a 1.5 ml microfuge tube, and centrifuged for 3 minutes at 11 000 g. The pellets were resuspended in 900 µL Buffer 1 (100 mM Tris pH 8.0, 500 mM sucrose, 0.5 mM EDTA, supplemented with cOmplete^TM^ Protease Inhibitor Cocktail (Roche, Mannheim, Germany) and incubated on ice for 5 min, after which the centrifugation was repeated. The pellets were resuspended in 400 µL of 1 mM MgCl_2_ with 100 µM N-ethylmaleimide and incubated on ice for 2 min, followed by centrifugation. The supernatants were carefully transferred to new tubes and stored as the periplasmic fractions. The pellets were resuspended in 1 mL Buffer 2 (50 mM Tris pH 8.0, 250 mM sucrose, 10 mM MgSO_4_, 100 µM N-ethylmaleimide, and cOmplete^TM^ Protease Inhibitor Cocktail), and centrifugation was repeated. The pellets were then resuspended in 750 µL Buffer 3 (50 mM Tris pH 8.0, 2.5 mM EDTA, 100 µM N-ethylmaleimide, and cOmplete^TM^ Protease Inhibitor Cocktail tablet) and disrupted using five cycles of 8 µm sonication for 40 s. The samples were kept on ice for 20 s between cycles. The samples were then centrifuged at 10 000 g for 5 min at 4 °C. The pellets were discarded and the supernatants were centrifuged again at 21 000 g for 30 min at 4 °C. Supernatants were transferred to new tubes and stored as cytoplasmic fractions. The final pellets were resuspended in 500 µL of Buffer 3 and stored as insoluble membrane fractions.

### Sample preparation for liquid chromatography-mass spectrometry LC-MS/MS

The samples were reduced with 10 mM dithiothreitol at 56 °C for 30 min followed by alkylation with 20 mM iodoacetamide for 30 min at room temperature in the dark. Proteins were precipitated overnight with ice-cold ethanol to a final concentration of 90% at −20 °C. The samples were centrifuged at 14 000 g for 10 min at 4 °C. The ethanol was removed and the samples were resuspended in 100 mM ammonium bicarbonate and sonicated using a Bioruptor (45 cycles, 30 sec on/30 sec off), followed by centrifugation at 14 000 g for 10 min. The supernatants were then transferred to new tubes. Protein concentration was determined using a NanoDrop (DeNovix DS-11) at 280 nm. Samples (30 µg) were digested with trypsin (Promega, Madison, WI, USA) at a ratio of 1:50 w/w (enzyme:proteins) overnight at 37 °C in a total volume of 100 µL. Digestion was stopped using 10 µL of 10% trifluoroacetic acid (TFA). C18 columns (UltraMicroSpin Column C18, SUM SS18V, capacity 3–30 µg, The Nest group Inc., South Borough, were used for sample cleanup according to the manufacturer’s instructions. The samples were dried using a Speed Vac and resolved in 2% ACN/0.1% TFA. Peptide concentration was measured using a NanoDrop (DeNovix DS-11) at 215 nm.

### Mass spectrometry acquisition

Liquid chromatography-mass spectrometry (LC-MS) was performed at the Swedish National Infrastructure for Biological Mass Spectrometry (Lund, Sweden). LC-MS detection was performed essentially as has been described previously [29]. Briefly, the samples were prepared and analysed on two separate occasions on a Tribrid Fusion mass spectrometer equipped with a Nanospray Flex ion source and operated in data-dependent acquisition (DDA) mode. The mass spectrometer was coupled with an EASY-nLC 1000 ultra-high-pressure liquid chromatography (UHPLC) pump (Thermo Fischer Scientific). One microgram of peptides per sample were concentrated on an Acclaim PepMap 100 C18 precolumn (75 μm × 2 cm, Thermo Scientific, Waltham, MA) and separated on an Acclaim PepMap RSLC column (75 μm × 25 cm, nanoViper, C18, 2 μm, 100 Å) at a temperature of 45 °C and a flow rate of 300 nL/min. A non-linear gradient of 0.1% formic acid in acetonitrile was used to elute the peptides (for details, see [29]). One full MS scan (resolution 120,000; mass range of 350–1350 *m*/*z*) was followed by MS/MS scans (resolution 30,000) with a 3 sec cycle time. Precursors with a charge state of 2–5 were included. The automatic gain control (AGC) target was set to 4 × 10^5^ for MS and 5 × 10^4^ for MS/MS, with injection times of 50 and 54 ms, respectively. The precursor ions were isolated with 1.2 *m*/*z* isolation window and fragmented using higher-energy collisional-induced dissociation (HCD) at a normalized collision energy (NCE) of 30. The dynamic exclusion was set to 45 s and the mass tolerance window to 10 ppm.

### Proteomics data analysis

The data from the mass spectrometer were analysed in Proteome Discoverer 2.5 against the *Achromobacter xylosoxidans* UniProt proteome (Proteome ID: UP000595052). Fully tryptic digestion was used, allowing for two missed cleavages. Carbamidomethylation (C) was set to static, and protein N-terminal acetylation and oxidation (M) to variable modifications. The mass tolerance for precursor ions was set to 10 ppm and for fragment ions to 0.02 Da. The protein false discovery rate (FDR) was set to 1%. Proteins identified by two or more unique peptides were considered relevant, whereas others were discarded. Proteomics data are presented in Supplementary Table S2. The proteins identified in the periplasmic fractions were log2-transformed and sum-normalized using Perseus (v2.0.2.0) to generate boxplots. The normalized data for the periplasmic fractions are presented in Supplementary Table S3. The mass spectrometry data were deposited in the ProteomeXchange [30] consortium via the MassIVE partner repository (https://massive.ucsd.edu/) with the dataset identifier PXD043024.

### Bacterial adhesion to bronchial epithelial cells

The adhesion of *A. xylosoxidans* isolates to human bronchial epithelial cells was studied using the human bronchial epithelial cell line BEAS-2B. The cells were cultured in RPMI medium (Invitrogen) with 10% (v/v) foetal bovine serum (FBS)(Invitrogen) supplemented with 100 U·mL^−1^ penicillin and 100 U·mL^−1^ streptomycin (Fisher Scientific). For adhesion experiments, the cells were seeded into a 12-well microtiter plate coated with 1% bovine collagen solution (PureCol, Advanced Biomatrix) and grown until > 95% confluence to minimize the available surface area for bacterial adhesion to plastic.

Overnight cultures of *A. xylosoxidans* were washed twice in PBS and resuspended to a concentration of 10^5^ colony forming units (CFU)/ml in RPMI with 10% FBS.

Bronchial epithelial cells were washed twice with PBS, and 1 ml of diluted bacterial solution was added to each well at a magnitude of infection (MOI) corresponding to 1:1 bacterial cells to human cells. Bacterial suspensions were also added to wells that did not contain cells, as a negative control for background binding. The plates were gently centrifuged for 5 min at 500 × g to bring the bacteria in closer contact with the cells, followed by incubation at 37 °C for 2 h. The wells were then washed twice with PBS to remove non-adherent bacteria, and the washed cells were detached by incubation with 100 μL of trypsin-EDTA (Sigma-Aldrich) at 37 °C for 3 min. Thereafter, 900 μL of RPMI medium with 10% FBS was added to deactivate trypsin. Cell suspensions from each well were plated on LB agar at 1:1 and 1:50 dilutions and incubated at 37 °C for 48 h, after which colonies were counted. Adhesion was measured as the fraction of bacteria remaining after the incubation and washing steps when compared to the stock solution, after subtraction of the colony count observed in wells with no epithelial cells.

### Iron metabolism assays

Iron-limited growth assays were conducted in ABTGC medium using 96 well microtiter plates (ThermoScientific). Briefly, overnight cultures from each isolate were diluted to OD_600_ = 0.1 and 15 µL was added to a total volume of 150 µL medium in three wells of the plates. Each isolate was grown in ABTGC medium with 0 µM, 10 µM, or 20 µM Fe^3+^ that was added immediately before the experiment started. Bacterial growth (OD_600_) was measured continuously for 20 h at 37 °C with shaking using a SpectraMax Multimode Reader (Molecular Devices). Growth data were analysed using Microsoft Excel (Microsoft), where the doubling time of each strain was calculated during exponential growth. The experiments were replicated three times for each isolate, and statistical significance was calculated using a two-tailed Student’s t-test between the mean doubling times of the isolates.

### Statistical calculations

Comparisons between groups were made using Student’s t-test. Statistical analyses were performed using SPSS version 25 (SPSS, Armonk, NY, USA) and GraphPad Prism (GraphPad Software, San Diego, CA, USA). A two-tailed *p* < 0.05 was regarded statistically significant. No correction for multiple testing was performed in the proteome comparisons of predicted Tat substrates between the wildtype and ∆tatC mutant.

## Results

### Putative tat substrates are abundantly found in *A.* *xylosoxidans* proteome

**Figure 1.**
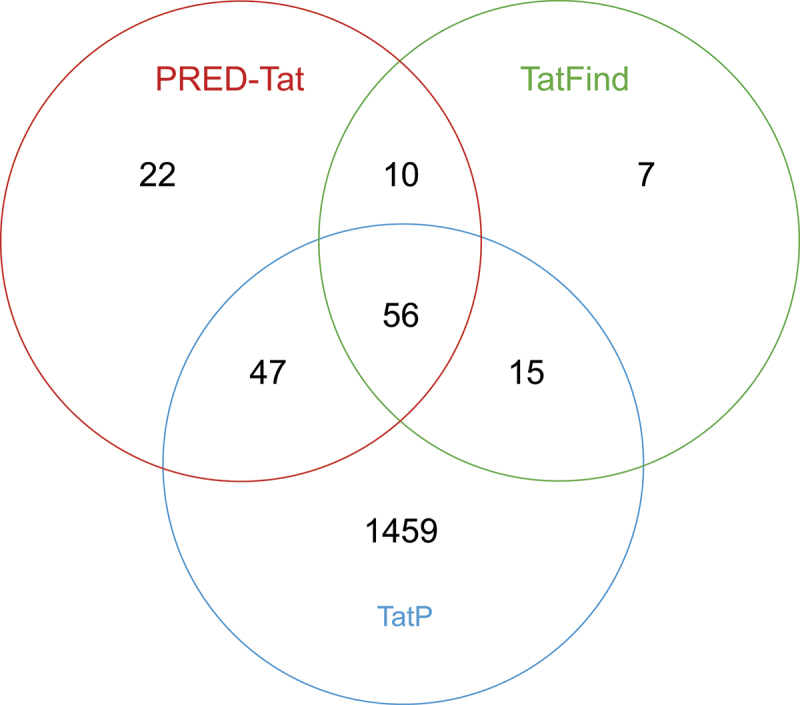
VENN diagram illustrating the results from three Tat substrate prediction algorithms; PRED-Tat (red), TatFind (green) and TatP (blue). The numbers inside the circles represent the number of putative Tat substrates identified by one, two, or three of the prediction tools.

Based on different experimental and *in silico* prediction methods, the Tat system is known to be involved in protein secretion in various bacterial species. To investigate the contribution of the Tat system to protein translocation in *A. xylosoxidans*, the *A. xylosoxidans* proteome was screened for putative Tat substrates. Three different *in silico* tools were used to predict the Tat signal peptides in the proteome: PRED-Tat, TatFind, and TatP. While PRED-Tat identified a total of 135 proteins, TatFind identified 88 and TatP identified 1577. To be considered a putative Tat substrate in this study, proteins had to be identified by at least two prediction tools. Using this definition, 128 predicted Tat substrates were identified in this study. Of these, 56 proteins were identified by all three prediction algorithms (Figure 1 and Supplementary Table S1).

To identify expressed putative Tat substrates, we performed a comprehensive proteomic characterization of *A. xylosoxidans*. The clinical *A. xylosoxidans* isolate LC1373 was grown in minimal medium and subsequently fractionated into membrane, periplasmic, and cytosolic fractions. The expressed proteome of each fraction was analysed using LC-MS/MS. We identified 2290 proteins with two or more unique peptides across all samples (Supplementary Table S2). Of the 128 putative Tat substrates identified in the *A. xylosoxidans* proteome using the prediction algorithms, 21 were identified as expressed proteins in the total dataset (Table 2). Eleven of the expressed proteins were identified by all three prediction tools (Table 2).

**Table 2.** Expressed putative Tat substrates identified in the *A. xylosoxidans* proteome.

Protein description	Uniprot accession	Identified by prediction tools (n)	Expressed in periplasm
ABC transporter substrate-binding protein	A0A0D6IGC9	3	Yes
ABC transporter substrate-binding protein	A0A0D6GVW1	3	Yes
ABC transporter substrate-binding protein	A0A0D6H3R4	3	Yes
ABC transporter substrate-binding protein	A0A0D6IPK9	3	Yes
Argininosuccinate lyase	A0A0D6FWL1	2	Yes
Argininosuccinate lyase	A0A0D6I447	2	Yes
Argininosuccinate lyase	A0A0D6IQY6	3	Yes
OmpH family outer membrane protein	A0A0D6HF24	2	Yes
BMP family protein	A0A0D6FDV3	3	Yes
BsSco	A0A0D6FIS4	2	Yes
D-alanyl-D-alanine carboxypeptidase	A0A7T2RFI8	2	Yes
Extracellular solute-binding protein	A0A0D6H151	3	Yes
Ferripyoverdine receptor	A0A0D6IKG8	3	Yes
Fimbrial protein	A0A0D6G5C4	2	Yes
Glutamine-binding periplasmic protein	A0A0D6HE12	3	Yes
Neu5Ac-binding protein	A0A0D6I797	3	Yes
Nitrous-oxide reductase	A0A0D6IAU8	3	Yes
Succinate dehydrogenase flavoprotein subunit	A0A0D6GP36	2	Yes
Twin-arginine translocation pathway signal	A0A0D6H5H3	2	Yes
Ubiquinol-cytochrome c reductase iron-sulphur subunit	A0A0D6FH58	2	Yes
Uncharacterized protein	A0A0D6HQ03	2	No

### Tat secretion is essential for host cell adhesion and uptake of iron by *A.* *xylosoxidans*

To investigate the role of Tat secretion in *A. xylosoxidans*, we generated an in-frame deletion mutant lacking the full sequence of the TatC subunit, without affecting the expression of other genes. This results in disrupted Tat secretion, as TatC is the key organizing subunit that binds TatA and B to form the Tat complex [31,32].

**Figure 2.**
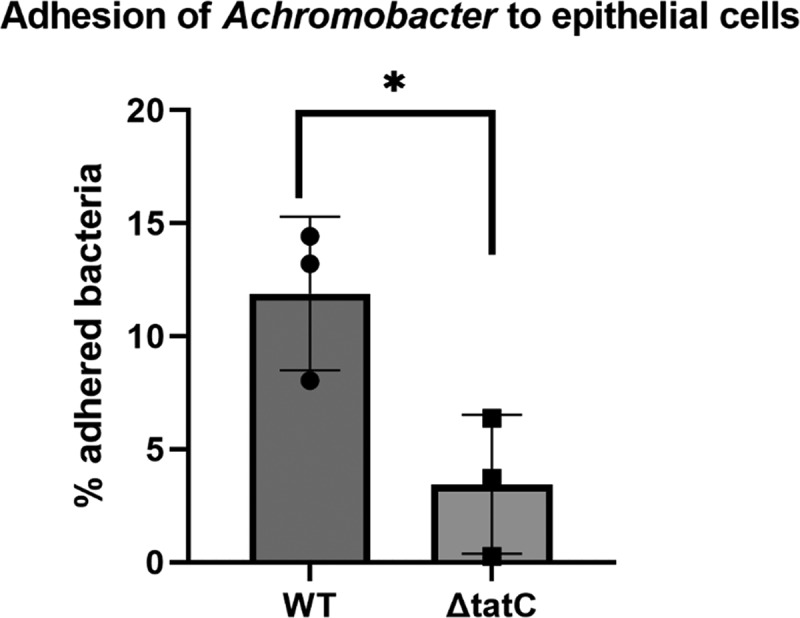
Percentage of remaining bacteria adhered to BEAS-2B lung epithelial cells after incubation and washing steps. Wild-type (WT) A. xylosoxidans adheres significantly better to epithelial cells than the ΔtatC mutant. The box plots illustrate the mean value of 3 repeats and the error bars the standard deviation. The dots represent individual values. Statistical comparison was performed using Student’s T-test. * = p < 0.05.

The ability to adhere to epithelial cells is key to the early establishment of lung infection. Therefore, we established an assay to assess bacterial adhesion to bronchial epithelial cells. Importantly, we found that the ΔtatC mutant showed significantly reduced adhesion to the bronchial epithelial cell line BEAS-2B compared to the parental wild-type strain (11.9% versus 3.5% binding, *p* < 0.05) (Figure 2).

**Figure 3.**
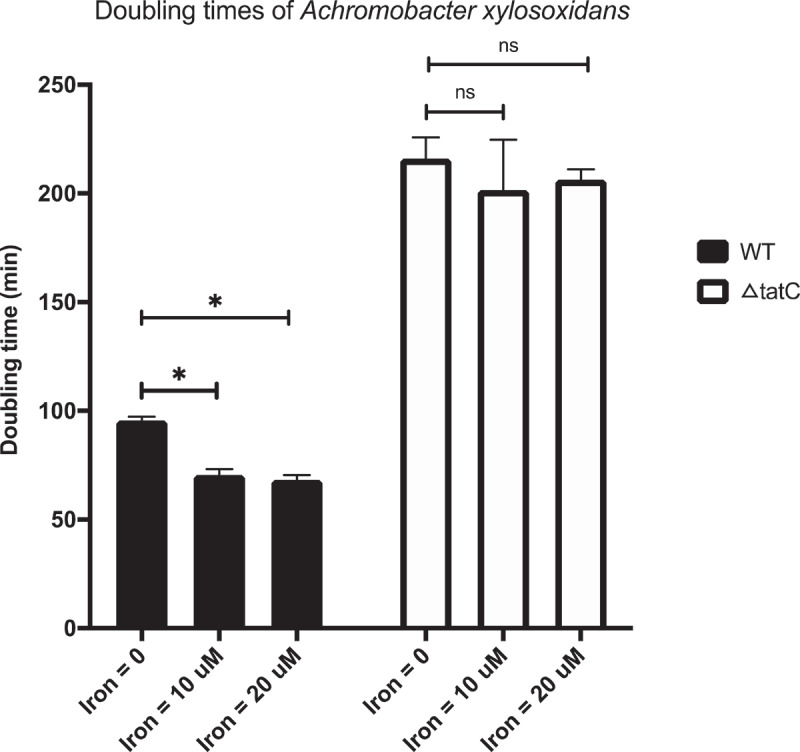
Growth rates of wild-type *A. xylosoxidans* and the ΔtatC mutant. The wild type and isogenic ΔtatC mutant were grown in minimal medium supplemented with different concentrations of iron (0, 10 or 20 uM, respectively). Optical density at 600 nm was monitored continuously to measure growth, and the doubling times were calculated during the exponential growth phase. The figure shows the mean growth rate expressed as doubling times (min) for the wild type (WT; black bars) and ΔtatC mutant (ΔtatC; white bars). Error bars represent SD. Statistical comparisons were made with Student’s T-test. * = p < 0.05, ns = non-significant.

While adhesion to the lung epithelium is important for the early establishment of infection, iron acquisition is essential for the establishment of persistent infections. Therefore, we investigated the role of Tat in iron acquisition by *A. xylosoxidans*. The ΔtatC mutant showed severely attenuated *in vitro* growth compared to the wild type, and the differences were even more pronounced under iron-limited conditions (Figure 3). While the wild-type strain grew significantly faster in the presence of iron compared to iron starvation, the growth rate of the isogenic ΔtatC mutant did not increase when iron was added to the medium. Taken together, our data suggest that the Tat system is important for adhesion to host cells, possibly via the translocation of membrane-bound peptides to the bacterial surface, and for iron acquisition in *A. xylosoxidans*.

### Identification of putative Tat substrates in the periplasm of *A.* *xylosoxidans*

To identify expressed predicted Tat substrates involved in the uptake of iron, wild-type and ΔtatC mutants were grown under normal and iron-limited conditions. The cells were then fractionated and analysed using LC-MS/MS, as described above, and the periplasmic proteomes of the wild-type and ΔtatC mutants were compared. A total of 2288 proteins were identified in the periplasmic fractions, of which 20 were putative Tat substrates (Table 2). All 20 Tat substrates were identified in both the wild-type and ΔtatC mutants, but there was significant contamination by cytosolic proteins in all samples, which may explain why Tat substrates were identified in the periplasm of the mutant (Supplementary Table S2). The summed raw MS intensities for all identified proteins did not differ between wild-type and ΔtatC mutants or between growth conditions (Supplementary Figure S1A), but proteins annotated to the cytosol were associated with the mutant (Supplementary Figure S1B, Supplementary Table S2), indicating leakage in the mutant cell wall or problems with the fractionation. Conversely, proteins annotated to the periplasm were associated with the wild-type (Supplementary Figure S1C, Supplementary Table S2).

**Figure 4.**
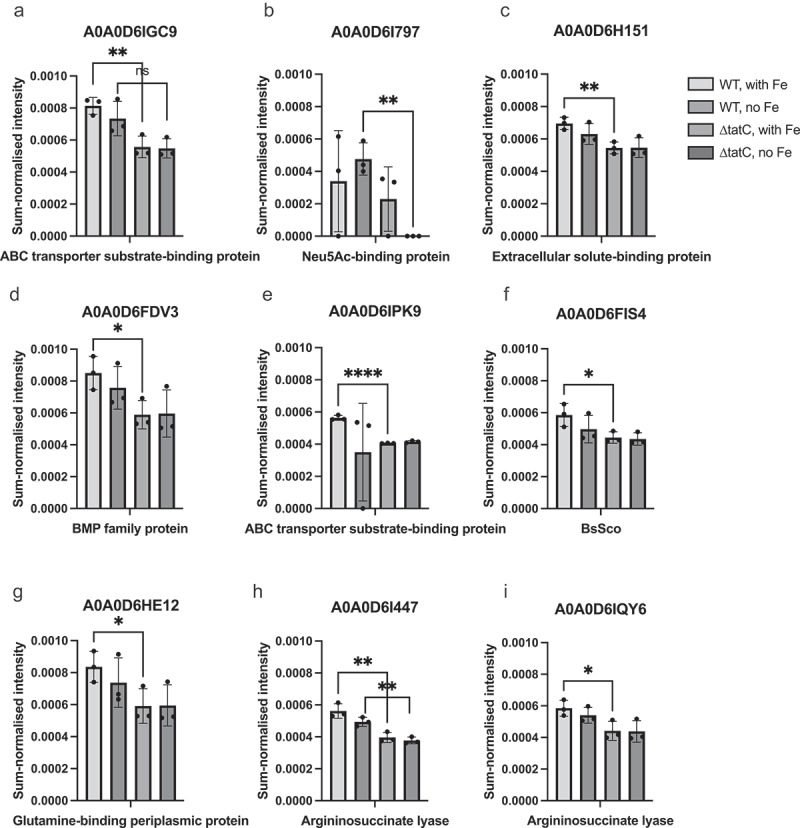
Expression of predicted Tat substrates in the periplasm of *A. xylosoxidans* during normal and iron limited conditions. The wild type (WT) and ΔtatC mutant were grown to mid-exponential phase in the absence or presence of iron. The cells were then separated into cytosolic, membrane and periplasmic fractions and the protein contents of the fractions were analysed with LC-MS/MS. The figure shows log2-transformed sum-normalized intensities for the putative Tat substrates expressed in the periplasmic fractions of the wild type and the ΔtatC mutant from three independent experiments (Supplementary table S3). The box plots show mean values and the error bars show standard deviation. The dots represent individual values. Comparisons between groups were made with Student’s T-test. * = p < 0.05, ** = p < 0.01, *** = p < 0.001, **** = p < 0.0001.

**Figure 5.**
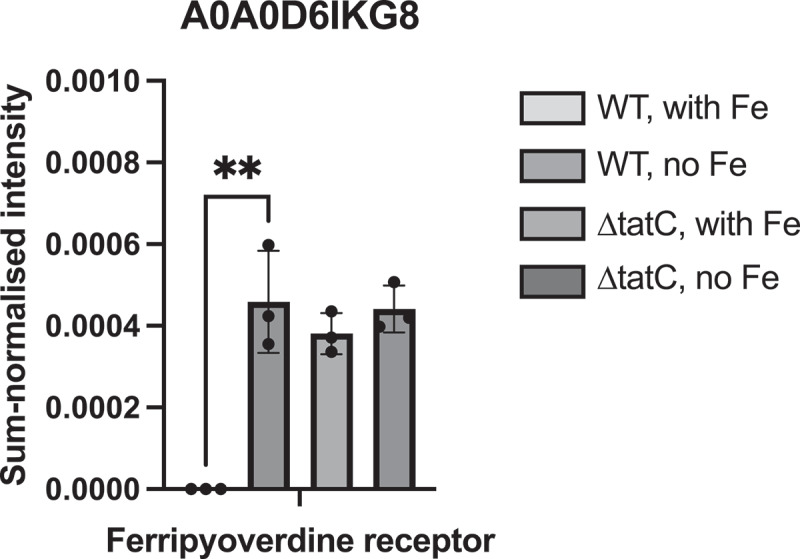
Expression of the predicted Tat substrate ferripyoverdine receptor. The wild type (WT) and the ΔtatC mutant were grown to mid-exponential phase in the absence or presence of iron. The cells were then fractionated and the protein contents of the periplasmic fractions were analysed with LC-MS/MS. The figure shows log2-transformed sum-normalized intensities for the putative Tat substrate ferripyoverdine receptor in the wild type and ΔtatC mutant from three independent experiments. The box plot shows the mean value and the error bars the standard deviation. The dots represent individual values. Comparisons between groups were made with Student’s T-test. ** = p < 0.01.

Nine of the 20 putative Tat substrates identified in the periplasm were significantly associated with the wild type (Figure 4), suggesting that these proteins were not properly translocated in the mutant because of TatC deletion. None of these proteins was significantly affected by iron starvation. Among the other 11 expressed putative Tat substrates, a ferripyoverdine receptor (FpvA) was completely absent in the wild type under normal conditions, but was expressed during iron starvation (Figure 5). The same protein was expressed in the ΔtatC mutant under both normal and iron-limited conditions, suggesting altered iron acquisition and that the mutant experiences iron shortage even when iron is present. The expression patterns of the remaining 10 putative Tat substrates identified in the periplasm did not differ in the presence or absence of iron or between the wild-type and ΔtatC mutants, except for the fimbrial protein A0A0D6G5C4, which was significantly associated with the ΔtatC mutant under iron-limited conditions (Supplementary Figure S2).

## Discussion

Tat secretion of folded proteins is used to varying extents by different bacterial species. While over 100 suggested Tat substrates have been identified in *Streptomyces* spp., important pathogens such as *Escherichia coli*, *Salmonella enterica* and *Campylobacter jejuni* express 15–30 predicted Tat substrates [12]. In this study, we identified 128 putative Tat substrates in the emerging CF pathogen *A. xylosoxidans* using three different prediction tools. In comparison, only 44 putative Tat substrates were identified in the *P. aeruginosa* PA14 proteome using the same prediction algorithms [22], suggesting that Tat secretion plays an important role in *A. xylosoxidans*.

In this study, we demonstrated that Tat secretion in *A. xylosoxidans* appears to be involved in cell growth, iron acquisition and adhesion to the host lung epithelium. In agreement with our findings, functional Tat secretion has been demonstrated to be required for iron acquisition in various pathogens [19,33,34]. The effects of Tat secretion on cell adhesion have not been previously described. However, it has been demonstrated to affect bacterial motility through the secretion of flagellar proteins [14,35], and flagella have also been implicated in cell adhesion [36].

To identify the Tat substrates involved in the phenotypic traits of the ΔtatC mutant, we performed extensive characterization of the expressed proteome of *A. xylosoxidans* wild-type and ΔtatC mutants grown with and without iron. Under these growth conditions, 20 putative Tat substrates were identified as proteins expressed in the periplasmic compartment. The only predicted Tat substrate that was upregulated during iron starvation was a ferripyoverdine receptor (FpvA) (Figure 5). In *P. aeruginosa*, FpvA is required for iron acquisition via the siderophore pyoverdine [37]. While Ochsner et al. described that the translocation of FpvA to the outer membrane is Tat-dependent [19], Voulhoux et al. found that FpvA was correctly located and functional in the cytoplasmic membrane of a Tat-mutant despite a Tat signal [38]. In the present study, FpvA was not expressed in the *A. xylosoxidans* wild type in the presence of iron, but was upregulated during iron limitation. In contrast, the protein was equally expressed under both growth conditions in the ΔtatC mutant, and there was no difference between the wild type and ΔtatC mutants during iron starvation (Figure 5). This finding supports previous reports that FpvA is not a true Tat substrate. Alternatively, the results may be affected by the fractionation method, leading to leakage of FpvA from the cytoplasmic compartment in the ΔtatC mutant. If so, it could be speculated that the ΔtatC mutant experiences iron starvation, despite the presence of iron in the growth medium. Consequently, the bacterium upregulates the expression of FpvA, which then becomes trapped in the cytosol due to defective Tat translocation. We observed substantial contamination of cytosolic proteins in all periplasmic fractions, and leakage was more pronounced in the ΔtatC mutant (Supplementary Figure S1). This is an important limitation that may interfere with the results, and the data must therefore be interpreted with caution. Interestingly, Tat is important for the outer membrane integrity in *E. coli* [39]. Similarly, tat mutants of *Salmonella* are attenuated due to envelope effects and failure to translocate the N-acetylmuramoyl-L-alanine amidases AmiA and AmiC and the cell division protein SufI [40]. Thus, the increased cytosolic leakage observed in the ΔtatC mutant might be caused by impaired membrane integrity due to a defect in the Tat system.

None of the nine putative Tat substrates associated with the wild type (Figure 4) could be linked to cell adhesion or iron acquisition. Even so, many of the proteins are involved in key cellular processes and may contribute to the attenuated phenotype of the ΔtatC mutant. For example, argininosuccinate lyases are key enzymes in the biosynthesis of arginine in both eukaryotic and prokaryotic organisms [41], and ATP-binding cassette (ABC) transporters are important for the uptake of a broad range of substrates, including iron, in bacteria [42]. However, none of the ABC transporters with a Tat signal peptide was affected in their expression by iron limitation. Importantly, it should be noted that phenotypes previously ascribed to Tat secretion seem to involve complex cellular processes, and no particular Tat substrate has often been linked to the observed phenotype [11]. In general, the annotation and knowledge of *Achromobacter* proteins is poor, and it is possible that some of these proteins are involved in processes that we currently have no knowledge of. In addition, it cannot be ruled out that other proteins important for iron acquisition require Tat secretion, even in the absence of a Tat signal. For example, proteins lacking the Tat signal peptide can co-translocate with Tat substrates and be transported through the Tat system as heterooligomeric complexes [12]. Moreover, it has been demonstrated that atypical Tat substrates can utilize the Sec machinery for the initial insertion, followed by an internal Tat signal to direct the remaining peptide via the Tat system [43,44]. An example of such a protein is the ferric citrate regulator FecR in *E. coli*, which is involved in iron acquisition [45]. Tat substrates with internal signal peptides were not detected using the prediction algorithms used in this study.

In conclusion, we demonstrated that the CF pathogen *A. xylosoxidans* expresses a large number of predicted Tat substrates and that Tat secretion may be important for cell growth, iron acquisition, and cell adhesion. Given the diversity of substrates secreted by the Tat system, *A. xylosoxidans* may utilize this secretion system to facilitate persistence under the stressful conditions of the CF airway, where bacteria are exposed to a myriad of environmental stressors, including immunological host factors, nutrient limitation, and varying oxygen availability [6]. Importantly, this first study involving a clinical isolate of *A. xylosoxidans* together with genetic tools and relevant infection models, has laid the foundation for future studies on the mechanisms underlying the establishment of persistent *A. xylosoxidans* lung infections in people with CF.

## Supplementary Material

Supplementary Figures Khademiclean.docx

Supplementary Table S1.xlsx

Supplementary Table S2.xlsx

Supplementary Table S3.xlsx

## Data Availability

The mass spectrometry data are openly available in the ProteomeXchange consortium via the MassIVE partner repository (https://massive.ucsd.edu/) with the dataset identifier PXD043024. All other data supporting the findings of this study are available within the article and its supplementary materials.
