# Supplementary material for: The twin-arginine translocation system is vital for cell adhesion and uptake of iron in the cystic fibrosis pathogen Achromobacter xylosoxidans
Source: Virulence. 2023 Nov 16;15(1):2284513. doi: 10.1080/21505594.2023.2284513 (PMC11533796; doi:10.1080/21505594.2023.2284513)
Supplement: Supplementary Figures Khademiclean.docx [file KVIR_A_2284513_SM3420.docx]

Supplementary Fig1

**Supplementary fig1:** Protein expression in the periplasmic compartment of *A. xylosoxidans* wildtype (WT) and the TatC mutant (ΔtatC). Bacteria were grown to mid-exponential phase in the absence or presence of iron. Cells were then fractionated and the protein contents of the periplasmic fractions were analysed with LC-MS/MS. Sub-cellular localisations for the identified proteins were determined based on annotations in UniProt (Supplementary table 2). The experiment was done in three independent replicates. The figure shows raw protein intensities for proteins identified in the periplasm of the wildtype and ∆tatC mutant. **(A)** The box plots show total raw intensities for all identified proteins in the periplasmic fractions. The number of identified proteins were 1991 in wt with iron, requiring an identification in one of the three replicates, 2120 in wt without iron, 2248 in ∆tatC mutant with iron and likewise 2248 in ∆tatC mutant without iron. **(B)** shows raw intensities for proteins annotated to the cytosol according to UniProt (n=262 in wt with iron, n=278 in wt without iron, n= 283 in mutant with iron and n=282 in mutant without iron). **(C)** shows raw intensities for proteins annotated to the periplasm according to UniProt (n=91 in wt with iron, n=91 in wt without iron, n= 87 in mutant with iron and n=87 in mutant without iron). Statistical comparisons were made using Student’s T-test. ** = p<0.01.

Supplementary Fig 2

**Supplementary fig 2:** Expression of predicted Tat substrates in the periplasm of *A. xylosoxidans* during normal and iron limited conditions. The wildtype (WT) and ∆tatC mutant were grown to mid-exponential phase in the absence or presence of iron. Cells were then separated into cytosolic, membrane and periplasmic fractions and the protein contents of the fractions were analysed with LC-MS/MS. The figure shows log2-transformed sum-normalised intensities for the putative Tat substrates expressed in the periplasmic fractions of the WT and ∆tatC mutant from three independent experiments. The protein intensities have been normalised across the different periplasmic fractions and expressed as normalised values in % of the total protein content. The box plots show the mean value and error bars the standard deviation. The dots represent individual values. Comparisons between groups were made with Student’s T-test. *** = p<0.001.
